# An open source web application for distributed geospatial data exploration

**DOI:** 10.1038/sdata.2019.14

**Published:** 2019-02-12

**Authors:** Patrick A. Curry, Nils Moosdorf

**Affiliations:** 1 Leibniz Centre for Tropical Marine Research (ZMT), Fahrenheitstraße 6, 28359 Bremen, Germany

**Keywords:** Geography, Research data, Research management

## Abstract

The number of online data repositories is growing and they are becoming increasingly difficult to navigate. Data are scattered among different repositories, or hidden on personal or institutional servers. To access data, users must search extensively and rely on site-specific tools. These hurdles substantially inhibit data findability and accessibility; in particular, those in the long tail of data. We developed an open source web application, Spatial Data Hub, that is a geospatial data index, connected to remote Internet sources. It allows simultaneous display and comparison of disparate datasets on a single map. It aims to promote all data equally and provide the flexibility to connect to any storage system, effectively making long-tail datasets as visible as those in large, established repositories. Its low barrier of entry allows scientists and organizations to easily add data throughout the research process; enhancing transparency, openness and reproducibility. This flexibility and functionality makes Spatial Data Hub a novel platform for researchers to promote their work, develop new hypotheses and create new collaborations.

## Introduction

The FAIR data principles^1^ and the TOP guidelines^2^ are a blueprint for high quality scientific data production in the current research environment. They promote scientific transparency and openness, and they promote good stewardship of data, analytical methodology and source code. In response to these initiatives and increases in data production, there are now thousands of online repositories, for example, re3data.org offers information on over 2000 repositories^3^ and OpenDOAR contains more than 3500^4^. The upsurge in available storage locations is a step forward for open science, but the data storage environment is becoming increasingly disparate and difficult to navigate. There need to be universal data exploration tools that can connect to repositories housing particular types of data. Accordingly, we describe a plan and the preliminary development of Spatial Data Hub, which is a centralized, extensible visualization tool that can connect to diverse, distributed repositories holding spatial data.

**Figure 1:**
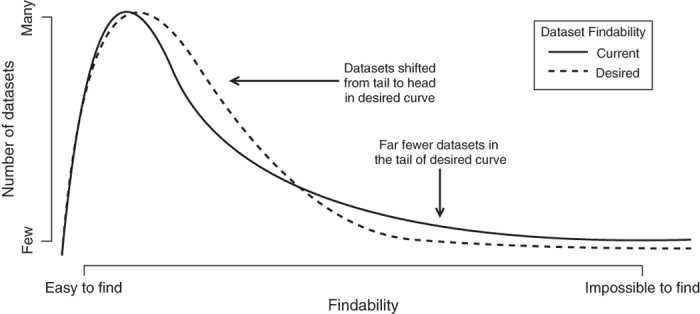
Expected effect of the presented application to dataset findability. Graphic is not to scale.

Large-scale data (e.g. satellite images, oceanic buoy observations) are generally kept in large, easy to find, centralized repositories. However, the data and metadata produced in daily research activities and small research projects often end up lost or in difficult to find locations. These are referred to as “long-tail” data^5^. They likely make up a high proportion of the data generated by researchers, yet they remain undiscovered after initial production and analysis^6,7^. In addition to these data, negative results typically do not get published^8^, despite the probability that they would aid in the development of a particular field. Boosting the findability of these long-tail data would increase scientific transparency, reduce redundant research and further scientific development. This would effectively concentrate more data on the highly findable end of the curve and reduce the long tail (Fig. 1).

Data for Earth system variables (e.g. weather station data^9^) are plentiful, but tend to be measured, stored and made available by the United States, Australia and European government agencies^10^. In less developed nations, data findability is inhibited by non-digital data storage^11^, out of date datasets, incomplete documentation, lack of data storage infrastructure and lack of funding for infrastructure^12^. Many of these developing nations are in tropical regions and have high population densities. Due to lacking data, these locations are not sufficiently represented in the training of global change models or other global scale analyses. These areas often coincide with strong changes predicted by the models^13^. Providing data from these areas would produce more accurate and dependable model results. To improve this situation, the data that have been collected in the field need to be made findable and available.

FAIR data^1^ and the TOP guidelines^2^ underpin scientific transparency^14^ and research reproducibility^15^ by encouraging publication of all results, including null results. They also stipulate persistent storage of data and metadata, data stored in machine readable formats and publication of research materials and analytical methods, especially computer code. A number of papers published in the last decade revealed difficulties in scientific reproducibility across disciplines^16–19^. Journal and funding agencies have made policy changes on data availability and analytical methodology that reflect the sentiment expressed in these papers^14,20–22^. Researchers are now encouraged to make their data, software code and statistical analysis scripts available for public use^23^. Data and code availability aid scientific transparency by boosting robust research practice and acting as impetus for the development of more specialized repositories.

Despite the implementation of FAIR principles and TOP guidelines, finding and exploring the correct data for a particular field of research remains a challenge. The exploration process requires researchers to interface with numerous search engines, government sites and data repositories that range widely in ease of use and credential requirements. Discontinuance of site maintenance, loss of project funding, site redesign leading to reference rot^24^ and site death also inhibit data findability and accessibility. Data source indices and search engines are currently the best tools for finding data. OpenDOAR (https://v2.sherpa.ac.uk/opendoar/) and re3data (https://www.re3data.org/) provide links to repositories, DataONE (https://www.dataone.org/) provides links to specific repositories and datasets, and tools like Pangaea (https://www.pangaea.de/) and Data Dryad (https://datadryad.org/) store and publish datasets with permanent identifiers, i.e. Digital Object Identifiers (DOI). A comparison of the merits and limitations of these systems, as well as further examples are listed in Table 1. Many of these tools have limited capabilities for data visualisation and exploration. Here we present a tool to find and visualise spatial data independent of its disciplinary background. Spatial Data Hub allows users to view and handle many spatial datasets directly on a map.

## Results

### Application state

**Figure 2:**
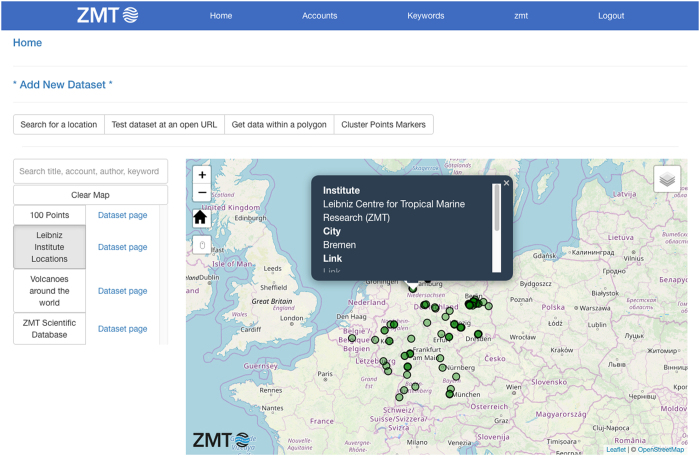
Appearance of the application’s demonstrator prototype available at www.spatialdatahub.org.

The Spatial Data Hub proof of concept is available at https://www.spatialdatahub.org. It features an attractive map centred interface (Fig. 2) and responsive design that allows users to comfortably view the website on screens of different sizes. A function bar and scrollable dataset list are located above and to the left of the map, respectively. The map background is switchable among a variety of tile options (openstreetmap, black and white, satellite view, etc.). Datasets can be displayed on the map as points, lines and polygons and have clickable popups displaying data properties. The main page acts as a template for person, account and keyword specific pages. The dataset registration page is a simple form asking the users to provide metadata, authentication credentials and a URL or file system link where the dataset can be found. Updating and removing dataset metadata and link information can be accomplished through similar form pages.

Spatial Data Hub’s core concept is dataset retrieval. It does this by making http requests to filesystems, sites and databases that hold the datasets. In the case that authentication credentials are not required, the client’s browser will make the request. If authentication is required, the Spatial Data Hub server will make the request and provide the credentials. Once the request has been sent and the dataset has been relayed to the client’s browser, the client can explore it and perform the various functions provided by Spatial Data Hub. Spatial Data Hub’s server is also capable of making requests to filesystems that require special authentication methods (at this stage: owncloud).

Datasets can be kept in a number of different formats (csv, tsv, kml, geojson) because Spatial Data Hub converts the datasets to the appropriate format for map display (geojson). Besides the standard zoom and data point popup functionality, Spatial Data Hub allows users to perform dataset URL tests, within dataset queries, point extraction, point clustering, location searches and dataset downloads. There is also a full screen function that allows maps with specific datasets (one or more) to be filtered and embedded into other web pages.

### Spatial Data Hub usage

Registering a dataset on Spatial Data Hub will affect different parties in different ways. For example, the Leibniz Centre for Marine Tropical Research (ZMT) has registered its own scientific database on the website. From the perspective of the ZMT, all of the research data its scientists have loaded to the database are advertised and available to anyone visiting the site. For the scientists that added their data to the database a new service has been provided without any work on their part. Any time they update their data or add more data to the ZMT’s scientific database, they will automatically be available on Spatial Data Hub through the dataset link. The third group, the outside users, are free to explore the data with the numerous functions the website provides. This service is transferable to any database providing data in the correct format (with correctly labelled fields representing latitude and longitude in decimal degrees) through a REST-API with cross origin resource sharing enabled.

A user can have many datasets in numerous formats stored across different systems (an issue common among scientific researchers). The only thing they need to do is to create a Spatial Data Hub account to view and explore them all in the same place. Furthermore, if the user needs to move the dataset from one storage location to another, Spatial Data Hub makes it easy to update the link information impacting the dataset itself. For a guide describing how to create an account and add datasets to it we have created a tutorial and made it available at the Spatial Data Hub GitHub page (https://github.com/spatialdatahub/spatialdatahub-tutorial).

## Discussion

Spatial Data Hub’s primary purpose is to increase the findability of long-tail geospatial data. It does this by giving datasets stored in small repositories and file systems the same level of promotion as those in governmental and large institutional systems. It does not require datasets to have persistent identifiers, which will encourage more users to share datasets from every step of the data collection process, including null results. Also, it allows researchers to make their work explorable as soon as it has been saved in a database or repository. It will be a boon to researchers not associated with large projects because it will make their datasets easier to find. Researchers can use it to advertise their work, and to use each other’s data. Papers with open datasets are cited more frequently^25^, and researchers who advertise their open datasets will likely receive a higher number of citations. Greater data availability will also feed into improved global modelling efforts and thus better-informed policies^26^. Making data available and findable can have numerous positive impacts, but the long-tail problem is not unique to data. It impacts many research disciplines. For example, certain tools, such as ESRI’s ArcGIS, are ubiquitously known in geospatially focused research and industry, but there are many less well known spatial tools that could be used for specific problems, for instance Geoda^27^, Geodetector^28^, SatScan^29^ and Sandwich Spatial Sampling and Inference^30^. This problem, and others, could be alleviated by the development of discipline focused online indices.

Spatial Data Hub is envisioned as a collaboration tool. As researchers increasingly use each other’s work they will necessarily establish lines of communication, thereby engendering new ideas and research directions. We do not currently know of a system that allows users to explore datasets from different repositories, and communicate through commenting or internal messaging systems. With the development of a messaging system and work check system scientists can engage in unofficial peer review activity by being able to see and work with datasets as soon as they are available. Because researchers will be able to upload preliminary and finished datasets alike, it will be easier to spot errors in the data through out the process. It will aid in the development of new hypotheses. Because users can display spatial data sets with different types of data on the same map, they will be able to develop the hypotheses based on visual assessments. A messaging system will make it easy to communicate these ideas to each other, thereby establishing new collaborations.

Future iterations of Spatial Data Hub will incorporate additional functionality on multiple levels. It can be further integrated with existing applications such as GitHub, Pangaea, DataOne, Fishbase and Zenodo, which will allow application specific data and metadata display with links that lead users to the other applications. To increase its ease of use and versatility, data request methods that traverse entire folders will be used to populate a user’s Spatial Data Hub account with datasets. Webhooks will allow it to listen for changes in a particular repository (for example GitHub) and automatically update to reflect new dataset files that have been added. This can be accomplished through contact with these other sites’ Application Programming Interfaces (APIs), which typically act as queryable data gateways. Integration will expand Spatial Data Hub’s role as an exploration tool to include data source discovery and promotion. New webhooks and API connections will also give clients the opportunity to utilize read and write capabilities. Datasets with proper licensing will be saveable in the user’s format of choice (e.g. geojson, csv, kml) to a client’s computer or to a repository offering webhook services. These features will be created so that they do not alter original data sources in any way.

More complex dataset transformations and spatial calculations, such as distance, area and centroid calculation will be incorporated with open source (turfjs) and proprietary (mapbox, ESRI) libraries. Other spatial analyses, like spatial autocorrelation, can be written into the client side code, but will require statistical expertise and a more complex user interface. The incorporation of time would also be extremely valuable and could allow visualisation of the changing phases of spatial datasets^31^. Users will be able to load data from their computer directly to the site and work on it. Spatial Data Hub will function on any computer with a web browser, effectively making it a cross platform solution. For work with relatively small datasets Spatial Data Hub could possibly be the easiest available option.

In addition to the site itself, the source code for Spatial Data Hub server^32^ and client^33^ are citable open access resources. The code base offers users and developers the opportunity to contribute directly to the project and shape the future of the site itself. In addition, developers can use the code base to learn how to create web applications as well as create their own projects. Ideally other projects could use Spatial Data Hub’s codebase as a foundation to build from. It would be easy to interchange spatial data with weather data, molecular data, or social data. The source code’s value comes from its ability to retrieve datasets and to create user-friendly interfaces for those datasets.

Data management is an ever-growing aspect of scientific research that engenders new method and tool development. Spatial Data Hub is aimed at simplifying data dispersion and manipulation by bringing a variety of functions together and reducing the number of necessary applications. It offers an extensible, open source code base that is written in some of the most widely used programming languages. We believe that Spatial Data Hub will be a valuable addition to the growing landscape of research oriented web tools.

## Methods

### Project code and development

The code was written following the test driven design technique in which the developer writes code tests before writing the actual code, and then they write the code so that it passes the tests. This ensures that everything that is written is planned out before hand, and it makes it easier for the developer to find errors as they add functionality. This technique was used in conjunction with continuous integration and deployment, meaning that once a feature is added, and all the tests pass, the actual web application will be automatically updated. Following continuous integration and deployment methodology allows continuous and incremental improvements to be incorporated into the application’s design, and it also makes responding to errors easier. The continuous integration system Spatial Data Hub relies on is Travis-CI, which is linked to Spatial Data Hub’s GitHub repository via webhooks and acts as a gatekeeper between the source code and the actual website’s server. The Spatial Data Hub site is hosted on Amazon Web Services using the Elastic Beanstalk development service.

To efficiently and securely deal with computational demands, the code has been split between the server and the client’s browsers. Fetching datasets that require authentication and handling sensitive information is performed on the server using the python based requests (http://docs.python-requests.org/en/master/) and cryptography (https://cryptography.io/en/latest/) libraries and the django web framework (https://www.djangoproject.com/). A postgresql database stores user information and dataset metadata on the server (https://www.postgresql.org/). Map display and GIS functionality are accomplished on the client’s browser with the javascript libraries, leaflet (https://leafletjs.com/) and Turf (http://turfjs.org/). Contributions to the project by outside developers are encouraged and guidelines are outlined in the project’s repository.

### Code availability

All of Spatial Data Hub’s code is open source and can be found at ref. 32,33. The project is covered by an MIT license that allows others freedom to do what they want with the code and protects the code’s authors.

### Data availability

No primary data were used for this study that could be made available.

## Additional information

**How to cite this article**: Curry, P. A. *et al*. An open source web application for distributed geospatial data exploration. *Sci. Data*. 6:190014 https://doi.org/10.1038/sdata.2019.14 (2019).

**Publisher’s note**: Springer Nature remains neutral with regard to jurisdictional claims in published maps and institutional affiliations.

## Figures and Tables

**Table 1:** Comparison of the merits and limitations of Spatial Data Hub along with five other systems.

Name	Description	Merits	Limitations
Antmapswww.antmaps.org	A map based front end and visualisation tool for the Global Ant Biodiversity Informatics database	• Open source code base• Impressive user interface• Easy to use	• Front end for a single database• Functionality purely focused on visualisation• Thematic niche focus
DataOnewww.dataone.org	A website that allows member repositories to share data and metadata links on a map background	• Access to a massive amount of data and metadata from many organizations• Provides a lot of dataset metadata including number of citations, views, and downloads• It is a large, well funded organization providing education and support	• Proprietary code base• Required to install and implement specific software• Limited to groups or organizations• Individual data points not visible on the map – links to metadata of datasets
Geojson.iowww.geojson.io	A GIS website providing dataset creation, display, editing and format conversion functionality	• Open source code base• Attractive and intuitive user interface• Highly featured	• Built to work with data, not focused on sharing• Limited support compared to larger programs
Nanooswww.nanoos.org	An interagency data visualisation and access portal focused on Northwestern United States	• Attractive user interface• High quantity data• Many apps that cover a wide variety of topics• Integrates space and time information	• Proprietary code base• Data visualisation for limited number of sources• Limited regional coverage
Pangaeawww.pangaea.de	A data publishing repository that puts datasets through quality control process, gives them DOIs and makes them publically available	• Permanent data storage, incl. DOI• Powerful data search function• Quality controlled• Hundreds of thousands of datasets available	• Proprietary code base• Data are manually curated before storage• Focused on single repository
Spatialdatahubwww.spatialdatahub.org	An open source web application that simultaneously displays geospatial datasets from disparate Internet sources on a single map	• Open source code base• Easily extensible• Can add and view multiple datasets from different sources	• No centralized repository• Front end developed for a specialized audience• Does not have the support system of the larger programs
